# Match demands and physical qualities of female athletes in Australian football, rugby union, rugby sevens, and rugby league: a scoping review

**DOI:** 10.5114/biolsport.2026.153306

**Published:** 2025-10-01

**Authors:** Riley J Brassington, Jocelyn K Mara, Nick Ball, Gordon Waddington, Kiera Paul, Julie Cooke

**Affiliations:** 1 University of Canberra Research Institute for Sport and Exercise (UCRISE), Canberra, Australia; 2 Discipline of Sport and Exercise Science, Faculty of Health, University of Canberra, Australia

**Keywords:** Anthropometry, Physical Performance, Collision Sports, Sport Science, Strength and Conditioning

## Abstract

This scoping review explored the physical qualities, and match demands of athletes participating at elite and sub-elite levels in four team-based collision sports played in Australia: Australian football, rugby union, rugby sevens, and rugby league. Fifty-nine studies were analysed to examine anthropometric traits, physical qualities, and match demands of female athletes across these sports. Comparisons were made between elite and sub-elite playing levels. A risk of bias and methodological assessment was also conducted. Findings suggest that elite athletes (as defined by the FTEM framework) in all sports, except rugby sevens, displayed greater height and body mass than sub-elite counterparts. Elite athletes in Australian football, rugby sevens, and rugby league demonstrated superior aerobic capacity, as measured by the Yo-Yo Intermittent Recovery Level 1 (IR1) test. Elite rugby union athletes were faster over 10-metre acceleration efforts and had higher average one-repetition maximum (1RM) bench press scores. The risk of bias assessment showed that 60% of studies had ‘unclear’ or ‘high risk’ of confounding due to uncontrolled or unreported contextual factors. All studies had ‘low risk’ of bias in assessor blinding and selective outcome reporting. These findings highlight the importance of specific anthropometric traits and physical qualities, such as greater body mass, lean mass, and aerobic capacity, as well as performance outcomes like relative running intensity and peak velocity, in distinguishing playing levels. These attributes can inform talent identification, enhance performance, and guide training interventions for sub-elite and elite female athletes.

## INTRODUCTION

Female participation in team-based collision sports has significantly increased in recent years, particularly in Australia, where sports such as Australian football, rugby union, rugby sevens, and rugby league have seen rapid growth in female engagement and the establishment of structured high-performance pathways [1–4]. Despite this surge in participation, sport science research has not kept pace. Recent reviews indicate that 71% of sports medicine studies focused on male participants, with only 6% involving female-only cohorts [5–7]. This gender disparity limits the development of evidence-based training and injury prevention strategies tailored to female athletes, in particular, exercise prescription often relies on male-centric data [3, 8]. Consequently, there is a need to develop data on the anthropometric traits and physical qualities of female athletes, as well as the match demands, experienced at all levels in these sports.

Collision sports are characterised by frequent, purposeful bodyto-body contact, that place high physical demands on athletes [9]. While each sport has its own match-play rules and characteristics that shape specific performance demands, they share several common physical requirements. Physical qualities such as strength, speed, aerobic capacity, and the ability to tolerate repeated high-intensity efforts are critical for performance [10–13]. Given the fact that most of the existing research has focused on male athletes, there is limited knowledge regarding which specific physical qualities differentiate performance levels among female athletes. A clearer understanding of these characteristics is required to support sport-specific training design, talent identification, and athlete development pathways that reflect the unique demands of female athletes.

The need to understand female specific characteristics and match demands is further supported by the reduced relative opportunity females have to train and develop athleticism as they progress throughout their career. A recent scoping review identified gender differences in practice specificity and training time allocation, highlighting challenges in designing effective talent development pathways tailored to female athletes [14]. Talent development pathways typically involve progression from youth and adolescent participation through to senior-level competition, where athletes may eventually reach elite or professional status. As seen in male collision sports i.e. Australian football [15], there are clear physical and performance differences between competition levels that allow coaches to structure informed physical preparation programs. Thus, identifying the key physical qualities that differentiate playing levels in female collision sports will enable strength and conditioning practices to be more precisely tailored to support athletes navigating the long-term athlete development pathway. For example, improvements in vertical jump performance have been associated with reduced 10-metre acceleration and 30-metre sprint times [16], while greater distances covered on the Yo-Yo IR1 test and faster sprint times have been linked to greater high-speed running distances during match play [11]; all of which may contribute to enhanced individual performance and overall team success [17].

To better understand the physical preparation practices required to enhance performance, an understanding of match demands is needed. Whilst information on match demands exists in some female sports at the elite level, limited data exists that compares match demands across different playing levels in female collision sports. Match demands can be quantified via internal load metrics, using ratings of perceived exertion (RPE), which has proved to be a valid measure of training load [18], and external workload metrics, such as total distance covered, high-speed running distance, and number of accelerations and decelerations. Recent studies in collegiate and professional female soccer players highlight a lack of research in subelite populations, which complicates the interpretation of external load profiles and limits the ability to design appropriate training strategies for developing athletes [19, 20]. Without this data, it is difficult to determine the physical intensities and demands experienced by athletes in lower tiers of competition, making it challenging to prescribe evidence-based training interventions that align with real world match requirements.

The aim of this scoping review is to systematically examine variations in anthropometric traits, physical performance qualities, and match demands, including external load measures such as distance covered and high-speed running (HSR), among female athletes in Australian football, rugby union, rugby sevens, and rugby league at both sub-elite and elite levels. By identifying key physical and matchrelated qualities that distinguish elite-level athletes from their subelite counterparts, this review seeks to inform evidence-based strength and conditioning practices and support long-term athlete development.

## MATERIALS AND METHODS

### Protocol

The review protocol was registered with the Open Science Framework (OSF) International and followed the PRISMA-ScR guidelines [21]. The FTEM framework, an evidence-based model that maps athlete development across foundational, talent, elite, and mastery stages, was used to define elite and sub-elite athletes [22]. Sub-elite athletes were in the ‘Talent’ stage, showing high performance potential (T1), verified talent (T2), practicing at higher levels (T3), and having professional support or breakthroughs (T4) [22]. Elite athletes were in the ‘Elite’ or ‘Mastery’ stages, with senior representation (E1), senior success (E2), or sustained success (M) at an elite level [22]. In professional sport, this includes playing at national or international levels with or without individual awards or team success. Athlete classification was clarified through competition levels, descriptions, or contextual information provided by the authors.

### Eligibility Criteria

Studies were included if they: 1) reported on female participants in Australian Football, Rugby Union, Rugby Sevens, or Rugby League; 2) were original peer-reviewed studies with full text in English; 3) provided data on anthropometric traits, physical qualities, and/or match demands; 4) involved participants aged 13 or older; 5) included sub-elite or elite athletes. Studies were excluded if they: 1) were review articles, case studies, dissertations, theses, or conference abstracts; 2) included only male participants; 3) involved participants younger than 13, as they would not be classified as developmental athletes in relation to the FTEM framework and would fall outside the age group for developmental/sub-elite squads; 4) investigated team-based court sports or non-collision sports; 5) were published before 1993 to ensure relevance, as the past three decades have seen significant advancements in the professionalisation of women’s sports and improvements in research reliability; 6) focused on concussion, mental health, or well-being.

### Information Sources

Studies were identified through comprehensive searches of multiple electronic databases. The systematic search was conducted on May 14, 2025, across MEDLINE, CINAHL, SCOPUS, SPORTDiscus, and Web of Science, with results limited to English-language publications. The search strategy was developed in consultation with an academic librarian experienced in systematic reviews and was piloted by two authors to refine and validate the selection of search terms.

### Search Strategy

The electronic database search involved using keywords to search the full text and citation records. Keywords within a group were combined using the Boolean operator ‘OR’, and then these groups were combined using ‘AND’. Proximity operators were used for some keywords to search for the root word. Selection of search terms was informed by the PICO (population, intervention, comparison, outcome) method. Five databases were searched by one reviewer (RB): ‘MEDLINE’, ‘CINAHL’, ‘SCOPUS’, ‘SPORTDiscus’, and ‘Web of Science’. The search string used was: ((women or female* OR woman OR girl*) AND (“match demand*” OR “activity profile*” OR “running demand*” OR “game demand*” OR “running performance” OR physical OR fitness OR “aerobic capacity” OR “repeated-sprint ability” OR anaerobic OR “countermovement jump” OR “vertical jump” OR strength OR power OR speed OR sprint* OR agility OR “physical qualities” OR recovery OR fatigue OR “muscle damage” OR training OR “training load”) AND (rugby OR league OR “super league” OR AFL or “Australian football league” OR “Australian rules” OR “collision sport*”)).

The search results were screened, and duplicates removed using Covidence systematic review management software (Veritas Health Innovation, Melbourne, Australia). Two reviewers (RB and JC) screened titles and abstracts to identify studies meeting the inclusion criteria. Full-text screening for suitability was conducted by RB and JC, with conflicts resolved through discussion. Descriptive data from included studies were extracted into a study-specific template in Microsoft Excel (Microsoft Corp., Redmond, WA). Data were independently extracted by RB and JC, who tested the template on a small subset of studies to ensure it captured necessary information and was easy to use. After extraction, RB and JC compared their results and discussed discrepancies to reach a consensus.

### Data Items

The following data were extracted from the included studies: study characteristics (author, year, study design), participant demographics (age, sex, competition level), sport (Australian Football, Rugby Union, Rugby Sevens, Rugby League), and outcome measures related to anthropometric traits (height, weight, body composition), physical qualities (strength, power, speed, endurance), and match demands (total distance covered, high-speed running, peak velocity). If the competition level (elite vs. sub-elite) was not explicitly stated, it was inferred from contextual information such as league level, team description, or training environment. No additional assumptions or simplifications were made.

### Risk of Bias

The risk of bias was evaluated independently by two authors (RB and JC), with disagreements reanalysed. If consensus was not reached, a third author (JM) made the final decision. The Risk of Bias Assessment Tool for Nonrandomised Studies 2 (RoBANS 2) was used, demonstrating acceptable feasibility, moderate reliability, and construct validity [23]. The tool comprises eight domains (Comparability of Target Group, Target Group Selection, Confounders, Measurement of Intervention/Exposure, Blinding of Assessors, Outcome Assessment, Incomplete Outcome Data, Selective Outcome Reporting), classified as ‘low’, ‘high’, or ‘unclear’ risk of bias [23]. All studies were included regardless of their risk of bias. However, studies rated as having a ‘high risk of bias’ due to multiple ‘high’ or ‘unclear’ risks were noted and not used as leading statements in the results.

### Methodological Quality Assessment

The Modified Downs and Black [24] evaluation scale was used to assess the methodological quality of the included studies by two authors (RB and JC). Of the total 27 criteria, 12 were used according to the study’s design (i.e., descriptive) as observed with similar systematic and scoping reviews [20, 25–27].

### Synthesis of Results

Data from the included studies were extracted and organised using a study-specific template in Microsoft Excel. Key variables (anthropometric traits, physical performance measures, match demands) were grouped by sport type, playing level, and gender where applicable. Central tendencies (means, medians) and variability (ranges, standard deviations) were calculated for each variable. The data were summarised in tables and charts for group comparisons. Due to methodological differences, the synthesis focused on presenting findings narratively, ensuring clarity without statistical pooling of heterogeneous data.

## RESULTS

### Search Results

**FIG. 1:**
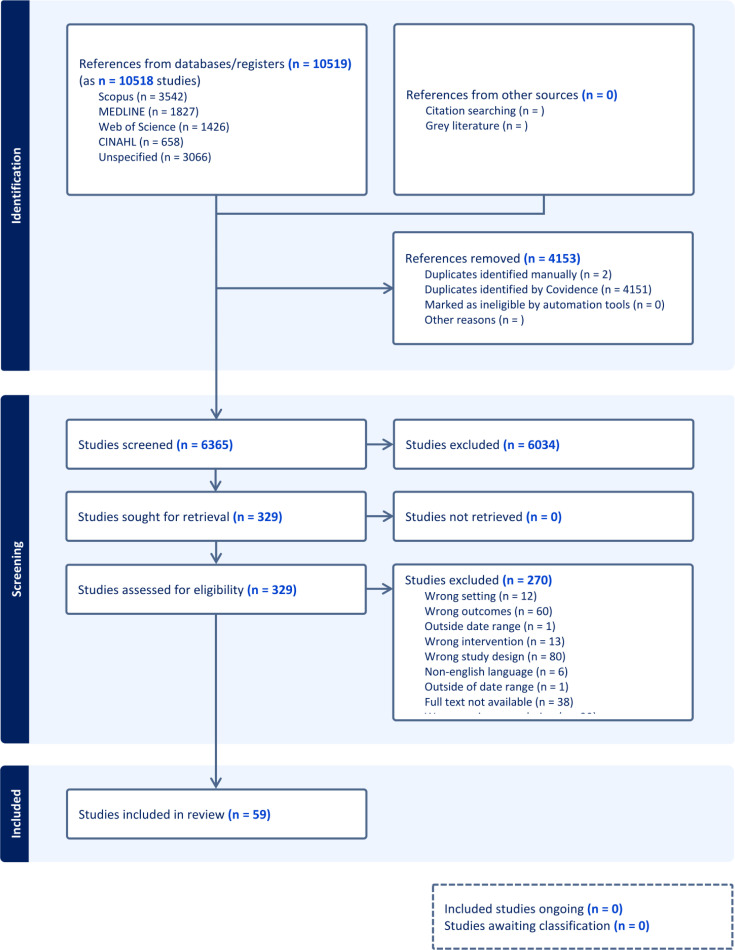
PRISMA flow diagram

Figure 1. depicts the PRISMA flow diagram of the research search and selection process. Of the 4153 studies reviewed, 59 met the inclusion criteria in this scoping review: 9 on Australian Football, 19 on Rugby Union, 18 on Rugby Sevens, 1 on Rugby League.

### Risk of Bias

Table 1 shows the risk of bias assessment. Overall, confounding variables were either ‘unclear’ or ‘high risk’ in 60% of the studies due to unreported or uncontrolled contextual factors (e.g., sleep, nutrition, training, climate). For studies reporting on match profiles, confounders were listed as ‘low’ where appropriate. The risk of bias in the measurement of *Intervention/Exposure* was ‘unclear’ in 6.7% and ‘high risk’ in 8.3% of the studies. All included studies had a ‘low risk’ of bias in the *Blinding of Assessors* and *Selective Outcome Reporting* assessment.

**TABLE 1:** RoBANS-2 – Risk of Bias Assessment Tool

Author (year)	Blinding of assessors	Comparability of target group	Confounders	Incomplete outcome data	Measurement of intervention/exposure	Outcome assessment	Selective outcome reporting	Target group selection
Agar-Newman et al. (2015) [87]	*Low*	*Low*	*Unclear*	*Low*	*Low*	*Low*	*Low*	*Low*
Agar-Newman et al. (2017) [45]	*Low*	*Unclear*	*Unclear*	*Low*	*Low*	*Low*	*Low*	*Low*
Alonso-Aubin et al. (2021a) [86]	*Low*	*Low*	*High*	*Low*	*Low*	*Low*	*Low*	*Low*
Alonso-Aubin et al. (2021b) [88]	*Low*	*Low*	*Low*	*Low*	*Low*	*Low*	*Low*	*Low*
Bicudo et al. (2025) [100]	*Low*	*Low*	*Unclear*	*Low*	*Low*	*Low*	*Low*	*Low*
Black et al. (2018a) [72]	*Low*	*Low*	*Low*	*Unclear*	*Low*	*Low*	*Low*	*Low*
Black et al. (2018b) [11]	*Low*	*Unclear*	*Unclear*	*Low*	*Unclear*	*Low*	*Low*	*Low*
Black et al. (2018c) [29]	*Low*	*Low*	*Unclear*	*Low*	*Low*	*Low*	*Low*	*Low*
Bradley et al. (2020) [43]	*Low*	*Low*	*Unclear*	*Low*	*Low*	*Low*	*Low*	*Low*
Brosnan et al. (2022) [54]	*Low*	*Low*	*Low*	*Low*	*Low*	*Low*	*Low*	*Low*
Busbridge et al. (2022) [12]	*Low*	*Low*	*Low*	*Low*	*Low*	*Unclear*	*Low*	*Low*
Callanan et al. (2021) [39]	*Low*	*Low*	*Unclear*	*Low*	*Low*	*Low*	*Low*	*Low*
Clarke et al. (2017) [46]	*Low*	*Low*	*Low*	*Low*	*Low*	*Low*	*Low*	*Low*
Clarke et al. (2018) [28]	*Low*	*Low*	*Low*	*Low*	*Low*	*Low*	*Low*	*Low*
Clarke et al. (2019) [30]	*Low*	*Low*	*Unclear*	*Low*	*Low*	*Low*	*Low*	*Low*
Couderc et al. (2023) [42]	*Low*	*Low*	*Unclear*	*Low*	*Low*	*Low*	*Low*	*Low*
Cummins et al. (2021) [110]	*Low*	*Low*	*Low*	*Low*	*Unclear*	*Low*	*Low*	*Low*
Cummins et al. (2023) [66]	*Low*	*Low*	*Unclear*	*Low*	*Low*	*Low*	*Low*	*Low*
Cummins et al. (2024) [67]	*Low*	*Low*	*Low*	*Low*	*Low*	*Low*	*Low*	*Low*
Curtis et al. (2022) [32]	*Low*	*Low*	*Unclear*	*Low*	*Low*	*Low*	*Low*	*Low*
Emmonds et al. (2020) [65]	*Low*	*Low*	*Low*	*Low*	*Low*	*Low*	*Low*	*Low*
Falk Neto et al. (2021) [89]	*Low*	*Low*	*High*	*Low*	*Low*	*High*	*Low*	*Low*
Flannery et al. (2024) [61]	*Low*	*Low*	*Unclear*	*Low*	*Low*	*Low*	*Low*	*Low*
Freitas et al. (2021) [97]	*Low*	*Low*	*Low*	*Low*	*Low*	*Low*	*Low*	*Low*
Gabbett et al. (2007) [60]	*Low*	*Low*	*Unclear*	*Low*	*High*	*Unclear*	*Low*	*Low*
Goodale et al. (2016) [57]	*Low*	*Low*	*Unclear*	*Low*	*Low*	*Low*	*Low*	*Low*
Goodale et al. (2017) [13]	*Low*	*Low*	*Unclear*	*Low*	*Low*	*Low*	*Low*	*Low*
Harty et al. (2021) [33]	*Low*	*Low*	*High*	*Low*	*Low*	*Low*	*Low*	*Low*
Heffernan et al. (2021) [90]	*Low*	*Low*	*Unclear*	*Low*	*Unclear*	*Low*	*Low*	*Low*
Hills et al. (2024) [99]	*Low*	*Low*	*Low*	*Low*	*Low*	*Low*	*Low*	*Low*
Imbert et al. (2023) [79]	*Low*	*Unclear*	*Unclear*	*Low*	*Low*	*Low*	*Low*	*Low*
Jennings et al. (2025) [78]	*Low*	*Low*	*Unclear*	*Low*	*Low*	*Low*	*Low*	*Low*
Jones et al. (2016) [58]	*Low*	*Low*	*Unclear*	*Low*	*Low*	*Low*	*Low*	*Low*
Lockie et al. (2016) [91]	*Low*	*Unclear*	*High*	*Low*	*High*	*High*	*Low*	*Unclear*
Loturco et al. (2021) [50]	*Low*	*Low*	*Low*	*Low*	*Low*	*Low*	*Low*	*Low*
Malone et al. (2020) [53]	*Low*	*Low*	*Low*	*Low*	*Low*	*Low*	*Low*	*Low*
Minahan et al. (2021) [57]	*Low*	*Low*	*Low*	*Low*	*Low*	*Low*	*Low*	*Low*
Misseldine et al. (2021) [98]	*Low*	*Low*	*Low*	*Low*	*Low*	*Low*	*Low*	*Low*
Muller et al. (2024a) [48]	*Low*	*Low*	*Unclear*	*Low*	*Low*	*Low*	*Low*	*Low*
Muller et al. (2024b) [51]	*Low*	*Low*	*Unclear*	*Low*	*Low*	*Low*	*Low*	*Low*
Newans et al. (2021) [63]	*Low*	*Low*	*Low*	*Low*	*Low*	*Low*	*Low*	*Low*
Nyman et al. (2022) [44]	*Low*	*Low*	*Unclear*	*Low*	*Low*	*Low*	*Low*	*Low*
Portillo et al. (2014) [56]	*Low*	*Low*	*Low*	*Low*	*Low*	*Low*	*Low*	*Low*
Posthumus et al. (2020) [80]	*Low*	*Low*	*Unclear*	*Low*	*Low*	*Unclear*	*Low*	*Low*
Quinn et al. (2020) [64]	*Low*	*Low*	*Low*	*Low*	*Low*	*Low*	*Low*	*Low*
Scantlebury et al. (2022) [62]	*Low*	*Low*	*High*	*High*	*High*	*Low*	*Low*	*Low*
Scantlebury et al. (2024) [59]	*Low*	*Low*	*Unclear*	*Low*	*Low*	*Low*	*Low*	*Low*
Sella et al. (2023) [101]	*Low*	*Low*	*Unclear*	*Low*	*Low*	*Low*	*Low*	*Low*
Sheppy et al. (2020) [40]	*Low*	*Low*	*Unclear*	*Low*	*Low*	*Low*	*Low*	*Low*
Suarez-Arrones et al. (2012) [55]	*Low*	*Low*	*Low*	*Low*	*High*	*Low*	*Low*	*Low*
Suarez-Arrones et al. (2014) [37]	*Low*	*Low*	*Low*	*Low*	*High*	*Low*	*Low*	*Low*
Thornton et al. (2022) [76]	*Low*	*Low*	*Low*	*Low*	*Low*	*Low*	*Low*	*Low*
Vescovi et al. (2015) [59]	*Low*	*Low*	*Unclear*	*Low*	*Low*	*Low*	*Low*	*Low*
Wijekulasuriya et al. (2024) [75]	*Low*	*Low*	*Unclear*	*Low*	*Low*	*Low*	*Low*	*Low*
Wilkinson et al. (2025) [31]	*Low*	*Low*	*Low*	*Low*	*Low*	*Low*	*Low*	*Low*
Woodhouse et al. (2021) [38]	*Low*	*Low*	*Unclear*	*Low*	*Low*	*Low*	*Low*	*Low*
Woodhouse et al. (2022) [41]	*Low*	*Low*	*Low*	*Low*	*Low*	*Low*	*Low*	*Unclear*
Yao et al. (2021) [35]	*Low*	*Low*	*Low*	*Low*	*Low*	*High*	*Low*	*Low*
Yao et al. (2024) [36]	*Low*	*Low*	*Unclear*	*Low*	*Low*	*Low*	*Low*	*Low*

### Methodological Quality Assessment

Using the Modified Downs and Black evaluation scale, studies ranged from 6 to 12 points out of a possible 12 (Table 2). One study scored 12/12, one scored 11/12, twenty studies scored 10/12, and twenty four scored 9/12, indicating that the majority (46) of the studies scored good-excellent for methodological quality.

**TABLE 2:** Modified Downs and Black Methodological Quality Assessment*

	Author, Year	1. Hypothesis / aims	2. Main outcomes	3. Charact-eristics of patients	4. Interventions	6. Main findings	7. Random Variability	10. Probability values	11. Participant (s) asked represents entire pop.	12. Participant (s) chosen represents entire pop.	16. Data Dredging made clear	18. Statistical tests appropriate	20. Main out-comes valid and reliable	Total Score /12
Australian Football (n = 9)	Black, 2018a [72]	Yes	Yes	Yes	Yes	Yes	Yes	No	Unable to determine	No	Yes	Yes	Yes	9
	Black, 2018b [11]	Yes	Yes	Yes	Yes	Yes	Yes	No	Unable to determine	No	Yes	Yes	Yes	9
	Black, 2018c [49]	Yes	Yes	Yes	Yes	Yes	Yes	No	Unable to determine	No	Yes	Yes	Yes	9
	Clarke, 2017 [46]	Yes	Yes	Yes	NA	Yes	Yes	No	Unable to determine	No	Yes	Yes	Unclear	7
	Clarke, 2019 [30]	Yes	Yes	Yes	Yes	Yes	Yes	No	Unable to determine	No	Yes	Yes	Yes	9
	Jennings, 2025 [78]	Yes	Yes	Yes	NA	Yes	Yes	No	Unable to determine	No	Yes	Yes	Yes	8
	Thornton, 2022 [76]	Yes	Yes	Yes	Yes	Yes	Yes	No	Unable to determine	No	Yes	Yes	Yes	9
	Wijekulasuriya, 2024 [75]	Yes	Yes	Yes	NA	Yes	Yes	Yes	Unable to determine	No	Yes	Yes	Yes	9
	Wilkinson, 2024 [31]	Yes	Yes	Yes	NA	Yes	Yes	No	Unable to determine	No	Yes	Yes	Yes	8

Rugby Union (n = 19)	Agar-Newman, 2015 [87]	Yes	Yes	No	Yes	Yes	Yes	Yes	No	No	Yes	Yes	Yes	9
	Alonso-Aubin, 2021a [86]	Yes	Yes	Yes	Yes	Yes	Yes	Yes	Unable to determine	No	Yes	Yes	Yes	9
	Bradley, 2020 [43]	Yes	Yes	Yes	Yes	Yes	Yes	No	Unable to determine	No	Yes	Yes	Yes	9
	Busbridge, 2022 [12]	Yes	Yes	Yes	Yes	Yes	Yes	Yes	Unable to determine	No	Yes	Yes	Yes	10
	Callanan, 2021 [39]	Yes	Yes	Yes	Yes	Yes	Yes	Yes	Unable to determine	No	Yes	Yes	Yes	10
	Curtis, 2022 [32]	Yes	Yes	Yes	Yes	Yes	Yes	No	Unable to determine	No	Yes	Yes	Yes	9
	FalkNeto, 2021 [89]	Yes	Yes	Yes	Yes	Yes	Yes	Yes	Unable to determine	No	Yes	Yes	Yes	10
	Harty, 2021 [33]	Yes	Yes	Yes	Yes	Yes	Yes	No	Unable to determine	No	Yes	Yes	Yes	9
	Heffernan, 2021 [90]	Yes	Yes	Yes	Yes	Yes	Yes	Yes	Unable to determine	No	Yes	Yes	Yes	10
	Imbert, 2023 [79]	Yes	Yes	Yes	Yes	Yes	Yes	Yes	Unable to determine	No	Yes	Yes	Yes	10
	Lockie, 2016 [91]	Yes	Yes	Yes	Yes	Yes	Yes	Yes	Unable to determine	No	Yes	Yes	Yes	10
	Nyman, 2022 [44]	Yes	Yes	Yes	Yes	Yes	Yes	Yes	Unable to determine	Yes	Yes	Yes	Yes	11
	Posthumus, 2020 [80]	Yes	Yes	Yes	Yes	Yes	Yes	Yes	Unable to determine	No	Yes	Yes	Yes	10
	Sheppy, 2020 [40]	Yes	Yes	Yes	Yes	Yes	Yes	Yes	Unable to determine	No	Yes	Yes	Yes	10
	Suarez-Arrones, 2014 [37]	Yes	Yes	Yes	Yes	Yes	Yes	Yes	Unable to determine	No	Yes	Yes	Yes	10
	Woodhouse, 2021 [38]	Yes	Yes	Yes	Yes	Yes	Yes	No	Unable to determine	No	Yes	Yes	Yes	9
	Woodhouse, 2022 [41]	Yes	Yes	Yes	Yes	Yes	Yes	No	Unable to determine	No	Yes	Yes	Yes	9
	Yao, 2021 [35]	Yes	Yes	Yes	Yes	Yes	Yes	Yes	Unable to determine	No	Yes	Yes	Yes	10
	Yao, 2024 [36]	Yes	Yes	Yes	NA	Yes	Yes	Yes	Unable to determine	No	Yes	Yes	Yes	9

Rugby Sevens (n = 18)	Agar-Newman, 2015 [87]	Yes	Yes	Yes	NA	Yes	Yes	Yes	Unable to determine	No	Yes	Yes	Yes	9
	Bicudo, 2025 [100]	Yes	Yes	Yes	NA	Yes	Yes	Yes	Unable to determine	No	Yes	Yes	Yes	9
	Brosnan, 2022 [54]	Yes	Yes	Yes	NA	Yes	Yes	Yes	Unable to determine	No	Yes	Unclear	Yes	8
	Clarke, 2017 [46]	Yes	Yes	Yes	No	Yes	Yes	No	Unable to determine	No	Yes	Yes	Yes	8
	Couderc, 2023 [42]	Yes	Yes	Yes	Yes	Yes	Yes	Yes	Unable to determine	No	Yes	Yes	Yes	10
	Freitas, 2021 [97]	Yes	Yes	Yes	Yes	Yes	Yes	Yes	Unable to determine	No	Yes	Yes	Yes	10
	Goodale, 2017 [13]	Yes	Yes	Yes	NA	Yes	Yes	Yes	Unable to determine	No	Yes	Yes	Yes	9
	Goodale, 2016 [57]	Yes	Yes	Yes	Yes	Yes	Yes	No	Unable to determine	No	Yes	Yes	Yes	9
	Hills, 2024 [99]	Yes	Yes	Yes	NA	Yes	Yes	Yes	Unable to determine	No	Yes	Yes	Yes	9
	Loturco, 2021 [50]	Yes	Yes	Yes	Yes	Yes	Yes	No	Unable to determine	No	Yes	Yes	Yes	9
	Malone, 2018 [53]	Yes	Yes	Yes	Yes	Yes	Yes	Yes	Unable to determine	No	Yes	Yes	Yes	10
	Middeldine, 2018 [98]	Yes	Yes	Yes	Yes	Yes	Yes	No	Unable to determine	No	Yes	Yes	Yes	9
	Muller, 2024a [48]	Yes	Yes	Yes	NA	Yes	Yes	Yes	Unable to determine	No	Yes	Yes	Yes	9
	Muller, 2024b [51]	Yes	Yes	Yes	NA	Yes	Yes	Yes	Unable to determine	No	Yes	Yes	Yes	9
	Portillo, 2014 [56]	Yes	Yes	Yes	NA	Yes	Yes	No	Unable to determine	No	Yes	Yes	Yes	8
	Sella, 2021 [101]	No	Yes	Yes	Yes	No	No	No	Unable to determine	No	Yes	Yes	Yes	6
	Suarez-Arrones, 2012 [55]	Yes	Yes	Yes	Yes	Yes	Yes	No	Unable to determine	No	Yes	Yes	Yes	9
	Vescovi, 2015 [49]	Yes	Yes	Yes	Yes	Yes	Yes	Yes	Unable to determine	No	Yes	Yes	Yes	10

Rugby League (n = 13)	Alonso-Aubin, 2021b [88]	Yes	Yes	Yes	Yes	Yes	Yes	Yes	Unable to determine	No	Yes	Yes	Yes	10
	Cummins, 2021 [110]	Yes	Yes	No	Yes	Yes	Yes	No	Unable to determine	No	Yes	Yes	Yes	8
	Cummins, 2023 [66]	Yes	Yes	No	Yes	Yes	Yes	Yes	Unable to determine	No	Yes	Yes	Yes	9
	Cummins, 2024 [67]	Yes	Yes	Yes	NA	Yes	Yes	Yes	Unable to determine	No	Yes	Yes	Yes	9
	Emmonds, 2020 [65]	Yes	Yes	Yes	Yes	Yes	Yes	Yes	No	No	Yes	Yes	Yes	10
	Flannery, 2024 [61]	Yes	Yes	Yes	NA	Yes	Yes	Yes	Unable to determine	No	Yes	Yes	Yes	9
	Gabbett, 2007 [60]	Yes	Yes	Yes	Yes	Yes	Yes	No	No	No	Yes	Yes	Yes	9
	Jones, 2016 [58]	Yes	Yes	Yes	Yes	Yes	Yes	Yes	Yes	Yes	Yes	Yes	Yes	12
	Minahan, 2021 [57]	Yes	Yes	Yes	Yes	Yes	Yes	Yes	Unable to determine	No	Yes	Yes	Yes	10
	Newans, 2021 [63]	No	Yes	Yes	Yes	Yes	Yes	Yes	Unable to determine	No	Yes	Yes	Yes	9
	Quinn, 2020 [64]	Yes	Yes	Yes	Yes	Yes	Yes	Yes	Unable to determine	No	Yes	Yes	Yes	10
	Scantlebury, 2022 [62]	Yes	Yes	Yes	Yes	Yes	Yes	Yes	Unable to determine	No	Yes	Yes	Yes	10
	Scantlebury, 2024 [69]	Yes	Yes	Yes	NA	Yes	Yes	Yes	Unable to determine	No	Yes	Yes	Yes	9

* Key: Criteria for Modified Downs and Black Methodological Quality Assessment

NA: Not Applicable
1. Is the hypothesis/aim/objective of the study clearly described? 2. Are the main outcomes to be measured clearly described in the Introduction or Methods section 3. Are the characteristics of the patients included in the study clearly described? 4. Are the interventions of interest clearly described? 6. Are the main findings of the study clearly described? 7. Does the study provide estimates of the random variability in the data for the main outcomes 10. Have actual probability values been reported (e.g. 0.035 rather than < 0.05) for the main outcomes except where the probability value is less than 0.001? 11. Were the subjects asked to participate in the study representative of the entire population from which they were recruited? 12. Were those subjects who were prepared to participate representative of the entire population from which they were recruited? 16. If any of the results of the study were based on “data dredging”, was this made clear? 18. Were the statistical tests used to assess the main outcomes appropriate? 20. Were the main outcome measures used accurate (valid and reliable)?

### Australian Football

Several studies have analysed the anthropometric traits (n = 8) (Table 3), physical qualities (n = 3) (Table 4), and match demands (n = 7) (Table 5) of female Australian football athletes across sub-elite and elite levels. This study found that athletes selected for matches exhibited greater aerobic fitness compared to non-selected athletes (Table 4), measured in the Yo-Yo IRT1 scores (712 m ± 251 m vs. 495 m ± 252 m, respectively).

**TABLE 3:** Anthropometric traits of female collision sport athletes

Study	Sport	Athletes (n)	Playing Level	Age (yrs)	Height (cm)	Weight (kg)	Sum Of Skinfolds (mm)	DXA	Playing Experience (years)
Black, 2018a [72]	Australian Football	49 Selected = 22 Non-selected = 27	Sub Elite Selected	23.2 ± 4.5	167.2 ± 5.0	67.8 ± 8.1			4.0 ± 2.8

			Sub Elite Non-selected	23.4 ± 4.9	167.9 ± 5.0	65.4 ± 9.0			2.1 ± 1.6
	
Black, 2018b [11]		22	Sub Elite	23.3 ± 3.8		62.5 ± 6.3			
	
Black, 2018c [49]		43	Sub Elite	24.3 ± 5.5	167.4 ± 4.3	66.5 ± 9.3			
	
Clarke, 2017 [46]		26	Elite	23.8 ± 7.6	171.2 ± 3.7	68.2 ± 7.3			
	
Clarke, 2019 [30]		59 (Elite = 23, Sub-elite = 36)	Elite	23.8 ± 7.6	171.2 ± 3.7	68.2 ± 7.3			

			Sub Elite	26.4 ± 4.5	168.4 ± 6.9	63.6 ± 6.8			
	
Thornton, 2022 [76]		28	Elite	24.1 ± 4.9	171.9 ± 6.7	68.3 ± 6.5			
	
Wijekulasuriya, 2024 [75]		15	Sub Elite	20.0 ± 3	1.65 ± 0.6	62.8 ± 10.3			
	
Wilkinson, 2024 [31]		22	Elite	25.0 ± 3.7					5.1 ± 2.69

Agar-Newman, 2015 [87]	Rugby Union	114	Sub Elite and Elite	17.91 ± 3.06		67.0 ± 6.91			
	
Alonso-Aubin, 2021a [86]		47 (17 = females)	Sub Elite	23.16 ± 1.38	163 ± 0.16	66.46 ± 2.39			
	
Bradley, 2020 [43]		129	Sub Elite	25 ± 5.62	169 ± 0.07	72.8 ± 6.04			
	
Busbridge, 2022 [12]		20	Sub Elite	23.7 ± 4.3	170 ± 6	79.1 ± 11.0			
	
Callanan, 2021 [39]		63	Sub Elite	F = 26.5 ± 4.4 B = 24.8 ± 4.1	F = 171.9 ± 6.9 B = 166.9 ± 5.1	F = 80.2 ± 8.4 B = 70.4 ± 6.5			
	
Couderc, 2023 [42]		33	Elite	25.6 ± 4.4	170 ± 9	73.1 ± 11.7			
	
Curtis, 2022 [32]		15	Elite	27 ± 5	169 ± 5	73.7 ± 9.6		BM (kg) – 74.9 ± 10.2 FM (kg) 21.0 ± 8.8 BMD (g · cm−2) 1.31 ± 0.06 LM (kg) 50.7 ± 3.9	
	
FalkNeto, 2021 [89]		17	Sub Elite	21.1 ± 2.6	168.5 ± 4.6	67–80.2 (± 7.2–15.4)			
	
Harty, 2021 [33]		101	Sub Elite	19.7 ± 1.6	166.4 ± 6.6	74.3 ± 15		BM (kg) – 74.3 ± 15 FM (kg) 19.8 ± 8.4 BMD (g · cm−2) 1.24 ± 0.09 LM (kg) 52.9 ± 7.7	
	
Heffernan, 2021 [90]		63	Elite	F = 28 ± 5 B = 26 ± 5	F = 171 ± 6 B = 166 ± 7	F = 83 ± 11 B = 73 ± 8			
	
Imbert, 2023 [79]		631 (n = 392 RU)	Elite	25 ± 3	169.4 ± 7	72.9 ± 12			
	
Lockie, 2016 [91]		8	Sub Elite	21.50 ± 1.77	160 ± 0.06	67.57 ± 10.49			
	
Nyman, 2022 [44]		29	Sub Elite	20.2 ± 2.5	166.5 ± 6.5	69.9 ± 10.1			
	
Posthumus, 2020 [80]		30	Elite	25.6 ± 4.3	171.3 ± 7.7	83.5 ± 13.9	111.3 ± 36.7 (8sf)	FM (kg) 20.3 ± 6.6 BMD (g · cm−2) 1.24 ± 0.08 LM (kg) 60.9 ± 7.8	
	
Sheppy, 2020 [40]		29	Elite	24 ± 3	167 ± 0.04	75.3 ± 10.8			
	
Suarez-Arrones, 2014 [37]		8	Elite	F = 26.6 ± 1.9 B = 27 ± 2.6	F = 173.8 ± 5.9 B = 170.3 ± 2.3	F = 76.8 ± 10.4 B = 68 ± 3.6	F = 99.3 ± 18.2 B = 89.3 ± 11.4			
	
Woodhouse, 2021 [38]		78	Elite	25 ± 4	170.6 ± 7.0	76.9 ± 9.8	F = 97.1 ± 14 B = 70.4 ± 6.4			

Woodhouse, 2022 [41]	Rugby Union	68	Elite	25 ± 4	170.6 ± 7.0	76.9 ± 9.8			
	
Yao, 2021 [35]		22	Sub Elite	26.9 ± 6.7	169.0 ± 5.9	75 ± 12.8		FM (kg) 17.7–26.9 LM (kg) 48.9–50.1
	
Yao, 2024 [36]		42	Sub Elite	F = 28.04 ± 5.98 B = 25.77 ± 3.87	F = 171.75 ± 7.98 B = 168.44 ± 4.67	F = 87.66 ± 12.60 B = 70.92 ± 4.40		F: FM (kg) 28.3 ± 11.69 LM (kg) 53.37 ± 5.1 B: FM (kg) 17.59 ± 5.0 LM (kg) 49.87 ± 4.05	

Agar-Newman, 2015 [87]	Rugby Sevens	23	Elite	22.8 ± 4.0	168.23 ± 5.3	69.36 ± 5.21	F = 84.4 ± 26.1 B = 95.0 ± 12.3		
	
Bicudo, 2025 [100]		21	Elite	25.4 ± 6.03					
	
Brosnan, 2022 [54]		54 (Elite = 21, Sub-elite = 33)	Elite	23.4 ± 3.5	168.6 ± 7	70.0 ± 5.4			

			Sub Elite	24.8 ± 4.0	168.8 ± 2.2	71 ± 9.5			
	
Clarke, 2017 [46]		33 (Sub Elite = 22, Elite = 11)	Elite		169 ± 0.02	68.6 ± 4.4	67 ± 14		

			Sub Elite		170 ± 0.07	70.4 ± 9.3	89 ± 20		
	
Couderc, 2023 [42]		18	Elite	24.2 ± 2.4	168 ± 4	67.6 ± 10.5			
	
Freitas, 2021 [97]		18	Elite	22.6 ± 4.6	166.7 ± 6.1	64.1 ± 7.2			
	
Goodale, 2017 [13]		24	Elite High-Playing Minutes	24.3 ± 3.1	167.7 ± 6.7	70.0 ± 4.9	86.8 ± 11.2		

			Elite Low-Playing Minutes	21.2 ± 4.3	168.8 ± 4.5	68.7 ± 5.7	91.6 ± 28.4		
	
Goodale, 2016 [57]		20	Elite	24.0 ± 3.6	168.4 ± 6.0	69.0 ± 5.0			
	
Hills, 2024 [99]		13	Elite	25.0 ± 5.0	175.0 ± 4	73.0 ± 4			
	
Imbert, 2023 [79]		631 (n = 239 R7)	Elite	24 ± 4	168.7 ± 6.9	65.7 ± 6.7			
	
Loturco, 2021 [50]		30 (Elite = 15, sub-elite 15)	Elite	23.9 ± 3.1	165 ± 0.07	66.4 ± 6.9			

			Sub Elite	22.9 ± 3.9	167 ± 0.09	66.3 ± 10.0			
	
Malone, 2018 [53]		27	Elite	24.4 ± 2.1	168 ± 7.1	67.9 ± 4.3			
	
Misseldine, 2018 [98]		12	Elite	F = 27.0 ± 2.5 B = 24.6 ± 4.7	F = 170.4 ± 3.3 B = 166.7 ± 5.3	F = 69.8 ± 2.0 B = 62.4 ± 4.4			
	
Muller, 2024a [48]		24	Sub Elite	16.88 ± 0.54	160.8 ± 6.66	62.69 ± 13.9			4.08 ± 2.55
	
Muller, 2024b [51]		31	Elite	25.74 ± 5.25	165.0 ± 0.06	63.64 ± 10.43	FM (kg) 13.12 ± 5.09 LM (kg) 50.48 ± 5.99 (7sf)		
	
Portillo, 2014 [56]		20 (Elite = 10, sub-elite = 10)	Elite	26.27 ± 4.05	166.7 ± 6.7	65.39 ± 5.01			

				Sub Elite	32.12 ± 6.40	167.4 ± 3.0	66.48 ± 5.38			
	
Sella, 2021 [101]		30	Sub Elite	22.0 ± 5.0	168 ± 0.05	69 ± 7			
	
Suarez-Arrones, 2012 [55]		12	Elite	27.8 ± 4.0	165.5 ± 6.2	63.7 ± 4.8			
	
Alonso-Aubin, 2021b [88]		87 (41 female)	Sub Elite	14.93 ± 2.76	163.0 ± 0.12	59.23 ± 12.66			

Flannery, 2024 [61]	Rugby League	79 Sub-Elite u19 = 45 Sub Elite Senior = 15 Elite = 19	Sub Elite u19	17.5 ± 0.6	167.7 ± 6.5	71.4 ± 12.8			

			Sub Elite Senior	24.3 ± 3.7	167.8 ± 5.1	70.9 ± 8.1			

			Elite	25.4 ± 4.0	167.9 ± 5.6	80.9 ± 14.6			
	
Gabbett, 2007 [60]		32	Elite	F = 18.9 ± 5.6 B = 18.9 ± 6.0	F = 169 ± 6.6 B = 166.1 ± 5.4	F = 75.5 ± 12.5 B = 64.7 ± 7.6	F = 141.2 ± 37.2 B = 114.8 ± 20.2		F = 3.0 ± 2.9 B = 2.9 ± 3.0
	
Jones, 2016 [58]		27	Sub Elite	F = 26.3 ± 6.4 B = 23.5 ± 4.1	F = 167.4 ± 6.8 B = 163.1 ± 4.0	F = 80.7 ± 14.3 B = 66.0 ± 7.3		BM (kg) -66–80.7 FM (kg) 18.2–26.9 LM (kg) 44.1–49.3 BMC (kg) 2.71–2.94		
	
Minahan, 2021 [57]		39	Elite	25 (21–27)	171.3 ± 7.7	68.4–84.3		FM (kg) 13.7–20.1 FFM (kg) 51.1–60.1
	
Newans, 2021 [63]		117	Sub Elite	26.8 ± 5.4	1.68 ± 0.07	76.7 ± 11.9			
	
Quinn, 2020 [64]		31	Elite						8.2 ± 2.6
	
Scantlebury, 2022 [62]		207 (182 sub elite, 25 Elite)	Elite	F = 23.7 ± 4.0 B = 23.8 ± 4.8	167.7 ± 5.2	73.0 ± 9.6			

			Sub Elite	F = 23.2 ± 5.8 B = 21.5 ± 4.8	164.5 ± 5.6	76.0 ± 15			
	
Quinn, 2020 [64]		24	Elite	F = 26.2 ± 4.6 B = 26.3 ± 5.8	F = 167.4 ± 4.1 B = 169.8 ± 4.6	F = 77 (71–82) B = 68 (62–73)		F: FM (kg) 22.495 (19.77–26.12) LM (kg) 50.41 (47.03–53.32) B: FM (kg) 17.41 (13.88–20.93) LM (kg) 47.17 (43.99–50.35)	

Key: F = Forwards; B = Backs; VSR = Vem Ser Rugby group; BRA = Brazilian youth rugby team; VJ = Vertical jump; CMJ = Countermovement jump

**TABLE 4:** Physical qualities of female collision sport athletes

Study	Sport	Athletes (n)	Playing Level	Age (y) Height (cm) Mass (kg)	10 m (s)	40 m (s)	MS momen-tum (kg/m/s)	1600 m Time T rial (s)	Yo-yo IRT1 (m)	1RM Front Squat (kg)	Relative Squat (kg/ BW)	1RM bench press (kg)	Standing Long Jump (cm)	Jump Height (cm)	Vmax (m/s)	Esti-mated ${\dot{\text{V}}\text{O}}_{2}$ peak (mL · kg · min)
Black, 2018a [72]	Australian Football	49 Selected = 22 Non-selected = 27	Sub Elite Selected						712 ± 251							

			Sub Elite Non-selected	23.3 ± 4.5 167.6 ± 5 66.4 ± 9.8					495 ± 252							
	
Wijekulasuriya, 2024 [75]		15	Sub Elite	20.0 ± 3 1.65 ± 0.6 62.8 ± 10.3						116 ± 30 (back squat, predicted 1RM)	1.85				7.44 ± 0.36	
	
Wilkinson, 2024 [31]		22	Elite	25.0 ± 3.7										33.2 ± 5.6 (Imp-Mom		

Agar-Newman, 2015 [87]	Rugby Union	114	Sub Elite	17.91 ± 3.06 NA 67.0 ± 6.91			474.04–519.72						191.0–221 ± 0.13		7.08–7.75	
	
Alonso-Aubin, 2021a [86]		47 (17 = females)	Sub Elite	23.16 ± 1.38 163. ± 0.16 m 66.46 ± 2.39						125.31 ± 8.23	1.89	37.81 ± 2.13				
	
FalkNeto, 2021 [89]		17	Sub Elite	21.1 ± 2.6		F = 6.7 ± 0.29 B = 6.28 ± 0.35										41.6–47.6
	
Heffernan, 2021 [90]		63	Elite	27 ± 5 168.73 ± 6.43 78.47 ± 9.70										24.4–28.7 (CMJ)		
	
Imbert, 2023 [79]		631 (n = 392 RU)	Elite	25 ± 3 169.4 ± 7 72.9 ± 12	1.87–1.92											
	
Lockie, 2016 [91]		8	Sub Elite	21.50 ± 1.77 160 ± 0.06 67.57 ± 10.49	2.05 ± 0.097								177 ± 0.18	42 ± 0.06 (VJ)		
	
Nyman, 2022 [44]		29	Sub Elite	20.2 ± 2.5 166.5 ± 6.5 69.9 ± 10.1											F = 5.29–7.76 B = 6.35–8.23	
	
Woodhouse, 2022 [41]		78	Elite	25 ± 4 170.6 ± 7.0 76.9 ± 9.8	1.81–1.96	5.5–6.12	369.1–461.0					61.1–86.3		32.1–39.6 (CMJ)		
	
Yao, 2021 [35]		22	Sub Elite	26.9 ± 6.7 NA NA	1.78–1.86 ± 0.06	5.83 (0.25)					88.50–92.33 (7.09–10.70)	NA	58.9–67.5 (7.79–9.2)	24.1–30.4 ± 5.72 (CMJ)		
	
Yao, 2024 [36]		42	Sub Elite	F = 28.0 ± 6.0 B = 25.8 ± 3.9 F = 171.8 ± 8.0 B = 168.4 ± 4.7 F = 87.7 ± 12.6 B = 70.9 ± 4.4										F = 25.0 ± 5.0 B = 32.3 ± 4.2	

Agar-Newman, 2015 [87]	Rugby Sevens	23	Elite	22.8 ± 4.0 168.23 ± 5.3 69.36 ± 5.21	F = 1.84 ± 0.04 B = 1.81 ± 0.04	F = 5.72 ± 0.12 B = 5.60 ± 0.14	F = 589.4 ± 34.4 B = 545.3 ± 32	F = 377 ± 25 B = 390 ± 28		F = 84.5 ± 5.8 B = 82.5 ± 11.3	F = 1.22 B = 1.19	F = 68.8 ± 7.1 B = 68.1 ± 7.2	F = 228 ± 9 B = 229 ± 11			
	
Bicudo, 2025 [100]		21	Elite	25.4 ± 6.03						109 ± 16.79	NA		2.14 ± 0.17			
	
Clarke, 2017 [46]		33 (Senior = 22, Elite = 11)	Elite	24.3 ± 3.1 167.7 ± 6.7 70.0 ± 4.9	1.76 ± 0.05	5.50 ± 0.16	565 ± 42		1702 ± 329					49.6 ± 3.8 (VJ)		
	
			Sub Elite	21.2 ± 4.3 168.8 ± 4.5 68.7 ± 5.7	1.82 ± 0.06	5.79 ± 0.17	556 ± 75		1058 ± 249					47.4 ± 5.5 (VJ)		
	
Goodale, 2016 [57]		24 (n = 12, 12)	Elite High Playing Minutes	22.8 ± 4.0 168.2 ± 5.6 69.4 ± 5.2	1.83 ± 0.05	5.66 ± 0.16	577.0 ± 29.1	374.5 ± 20.4		84.2 ± 7.9	1.21	68.4 ± 6.3	227.3 ± 9.0			

			Elite Low Playing Minutes		1.82 ± 0.03	5.66 ± 0.11	554.9 ± 47.1	393.5 ± 29.8		83.0 ± 9.6	1.20	62.2 ± 8.1	230.4 ± 11.0			
	
Imbert, 2023 [79]		631 (n = 239 R7)	Elite	24 ± 4 168.7 ± 6.9 65.7 ± 6.7	1.83 ± 0.08											
	
Loturco, 2021 [50]		30 (Elite = 15, sub-elite 15)	Elite	23.9 ± 3.1 1.65 ± 0.07 66.4 ± 6.9					1170.7 ± 252.3			65.2 ± 3.3				

			Sub Elite	22.9 ± 3.9 1.67 ± 0.09 66.3 ± 10.0					928.0 ± 249.0			40.3 ± 7.3				
	
Muller, 2024a [48]		24	Sub Elite	16.88 ± 0.54 160.8 ± 6.66 62.69 ± 13.9	VSR = 2.02 ± 0.12 BRA = 1.87 ± 0.08				VSR = 400.00 ± 186.76 BRA = 487.27 ± 156.79	VSR = 80.02 ± 16.98 BRA = 78.98 ± 18.79	VSR = 1.28 BRA = 1.26	VSR = 45.08 ± 6.80 BRA = 44.00 ± 8.19	VSR = 179.88 ± 15.15 BRA = 189.55 ± 16.93	VSR = 31.34 ± 5.41 BRA = 28.94 ± 3.66 (VJ)		
	
Muller, 2024b [51]		31	Elite	25.74 ± 5.25 165.0 ± 0.06 63.64 ± 10.43								58.64 ± 8.59	2.15 ± 0.15			
	
Sella, 2021 [101]		30	Sub Elite	22.0 ± 5.0 168.0 ± 0.05 69.0 ± 7.0	5.30 ± 0.18 (m·s−1) (n = 16)	6.72 ± 0.28 (m·s−1) (n = 16)		3.51 ± 0.27 (m·s−1) (n = 16)		90 ± 15 (BS, n = 26)	1.30	59 ± 10 (n = 28)		32.2 ± 4.1 (n = 20) (CMJ)		
	
Suarez-Arrones, 2012 [55]		12	Elite	27.8 ± 4.0 165.5 ± 6.2 63.7 ± 4.8													51.1 ± 3.6
	
Vescovi, 2015 [49]		29	Elite	NA NA NA						1160 ± 191 (1022–1239)							

			Sub Elite	NA NA NA						781 w ± 129 710–853)							
	
Alonso–Aubin, 2021b [88]	Rugby League	87 (41 female)	Sub Elite	14.93 ± 2.76 163.0 ± 0.12 59.23 ± 12.66						115.17 ± 41.42	1.94	45.85 ± 16.71				
	
Flannery, 2024 [61]		79 Sub-Elite u19 = 45 Sub Elite Senior = 15 Elite = 19	Sub Elite u19	17.5 ± 0.6 167.7 ± 6.5 71.4 ± 12.8											27.9 ± 4.7		

			Sub Elite Senior	24.3 ± 3.7 167.8 ± 5.1 70.9 ± 8.1											36.4 ± 6.5		

			Elite	25.4 ± 4.0 167.9 ± 5.6 80.9 ± 14.6 ±											35.7 ± 7.3		
	
Gabbett, 2007 [60]		32	Elite	18.9 ± 5.7 167.6 ± 6.1 70.1 ± 11.6	F = 2.04 ± 0.1 B = 1.96 ± 0.1	F = 6.59 ± 0.41 B = 6.33 ± 0.25									F = 35.1 ± 4.4 B = 35.7 ± 5.9 (vert jump)		F = 32.2 ± 4.4 B = 35.3 ± 3.4 (VJ)
	
Jones, 2016 [58]		27	Elite	24.7 ± 5.2 165.9 ± 5.4 73.4 ± 11.8	F = 2.01 ± 0.17 B = 1.87 ± 0.09	F = 6.59 ± 0.61 B = 6.13 ± 0.25			F = 610 ± 292 B = 728 ± 154						F = 24 ± 0.05 B = 29 ± 0.05		
	
Minahan, 2021 [57]		39	Elite	25.6 ± 4.3 171.3 ± 7.7 83.5 ± 13.9	F = 1.98 (1.93–2.04) B = 1.90 (1.84–1.95) A = 1.92 (1.85–2.00)										28.8–35.5 (CMJ)		42.7–47.2
	
Scantlebury, 2022 [62]		207 (182 domestic, 25 international	Elite	23.8 ± 4.4 167.7 ± 5.2 73.0 ± 9.6	1.93 ± 0.11	6.17 ± 0.34				763 ± 301.9 (mod)					29.1 ± 4.7 (CMJ)			

			Sub Elite	27.92 ± 5.37 164.5 ± 5.6 76.0 ± 15	2.07 ± 0.14	6.71 ± 0.61				522.7 ± 284.8 (mod)					24.8 ± 4.9 (CMJ)		
	
Scantlebury, 2024 [69]		24	Elite	F = 26.2 ± 4.6 B = 26.3 ± 5.8 F = 167.4 ± 4.1 B = 169.8 ± 4.6 F = 77 (71–82) B = 68 (62–73)													F = 40 (36 – 44) B = 41 (38 – 45)

* BS = Back Squat; B = Backs, F = Forwards, A = Adjustables; CMJ = Countermovement Jump; VJ = Vertical Jump; VSR = Vem Ser Rugby group; BRA = Brazilian youth rugby team;

**TABLE 5:** Match demands of female collision sport athletes

Study	Sport	Athletes (n)	Playing Level	Age (years) Height (cm) Mass (kg)	Matches (n)	TD (m)	RD (m/min)	MSR (m/min) (10–14.3 km/hr)	HSR (m/min) (14.4–20.99 km/hr)	VHSR (m/min) (> 21 km/hr)	Peak Velocity (m/s)
Black, 2018a [72]	Australian Football	49	Sub Elite	23.3 ± 4.5 167.6 ± 5 66.4 ± 9.8	14	4909–8018	72.7–108.9	18.7–36.5 (10.05–14.94 km/hr)	7.8–14.8 (> 14.94 km/hr)		
	
Black, 2018c [49]		43	Sub Elite	24.3 ± 5.5 167.4 ± 4.3 66.5 ± 9.3	6		94–109		13–14 (> 14.97 km/hr)		
	
Clarke, 2017 [46]		26	Elite	23.8 ± 7.6 171.2 ± 3.7 68.2 ± 7.3	7	4998–6255	102.1–128.4		17.2–28 (14.4–18.0 km/hr)	5.4–9.6 (18.1–20.0 km/hr)	6.75–7.11
	
Clarke, 2019 [30]		59	Elite	Elite 23.8 ± 7.6 171.2 ± 3.7 68.2 ± 7.3	20	5761–7234	95–126		9.8–18.8 (14.4–18.0 km/hr)	2.61–4.66 (18.1–20.0 km/hr)	6.75–7.14
		
			Sub Elite	Sub Elite 26.4 ± 4.5 168.4 ± 6.9 63.6 ± 6.8		6717–7222	94–106		10.6–13.1 (14.4–18.0 km/hr)	2.74–3.36 (18.1–20.0 km/hr)	6.67–6.94
	
Jennings, 2025 [78]		388	Sub Elite	NA	83	6500 – 7093	100 – 111		1052–1344 (14.4–19.99 km/hr) (TD)	48–85 (> 20.0 km/hr) (TD)	
	
Thornton, 2022 [76]		28	Elite	24.1 ± 4.9 171.9 ± 6.7 68.3 ± 6.5	7	6747 ± 1013	121 ± 12		1452 ± 415 (TD) (14.4–19.99 km/hr)	167 ± 120 (TD) (> 20.00 km/hr)	6.83 ± 0.44
	
Wilkinson, 2024 [31]		22	Elite	25.0 ± 3.7	13	6031–7568	118.4–137.9		442–1164 (TD) (16.9–20.87 km/hr)	28.9–44.0 (> 20.88 km/hr)	7.22 ± 0.36

Bradley, 2020 [43]	Rugby Union	129	Sub Elite	25 ± 5.62 169 ± 0.07 72.8 ± 6.04	14	4982 ± 917	54.8 ± 9.1		363 ± 218 (14.1–18 km/hr) (TD)	84 ± 87 (> 21.1 km/hr) (TD)	5.69–6.03
	
Busbridge, 2022 [12]		20	Sub Elite	23.7 ± 4.3 170 ± 6 79.1 ± 11.0	96	F = 5616 ± 809 B = 5829 ± 1022			F = 3.2 ± 0.5 B = 13.6 ± 0.7 (> 16.1 km/hr)		5.83–7.5
	
Callanan, 2021 [39]		63	Sub Elite	25.7 ± 4.4 169.4 ± 7.8 75.6 ± 10	12	5696 ± 822	67.9 ± 6.9	1,380 ± 383 (TD) (10.8–17.9 km/hr)	220 ± 156 (TD) (> 18.0 km/hr)	6.5 ± 0.7	
	
Couderc, 2023 [42]		33	Elite	25.6 ± 4.4 170 ± 9 73.1 ± 11.7	10	155 ± 21 (60 s peak intensity)	51 ± 23 (TD) (intercept) (> 16 km/hr)	7.6 ± 0.8
	
Sheppy, 2020 [40]		29	Elite	24 ± 3 167 ± 0.04 75.3 ± 10.8	8	5100–6100	78–84.2 (10–min epoch)	9.1–10.7 (10–min epoch)		
	
Suarez-Arrones, 2014 [37]		8	Elite	26.8 ± 2.3 172 ± 4.9 72.4 ± 9.4	1	5820 ± 512	553 ± 190 (TD) (14.1–18.0 km/hr)	105 ± 74 (TD) (18.1–20.0 km/hr)	73 ± 107 (TD) (> 20.1 km/hr)	F = 6.11 ± 3.5 B = 6.78 ± 0.8
	
Woodhouse, 2021 [38]		78	Elite	25 ± 4 170.6 ± 7.0 76.9 ± 9.8	53	3240–5283	62.1–72.9	16.8–46.0 (12.6–19.79 km/hr)	0.3–3.8 (> 19.8 km/hr)	

Bicudo, 2025 [100]	Rugby Sevens	21	Elite	25.4 ± 6.03	20	130–135	33–35 (> 18 km/hr)	
	
Brosnan, 2022 [54]		54 (Elite = 21, Sub-elite = 33)	Elite	Elite 23.4 ± 3.5 168.6 ± 7 70.0 ± 5.4	63	1500.2 (171.2)	94.3 (9.0)	444.7 (115.3) (TD) (11.88–21.5 km/hr)	77.0 (44.3) (21. 6 km/ hr – 26.99 km/hr)	19.8 (25.3) (TD) (> 27 km/hr)	7.7 (0.2)
	
			Sub Elite	Sub-Elite 24.8 ± 4.0 168.8 ± 2.2 71 ± 9.5		1463.5 ± 157.4	92.6 (8.1)	443.0 (108.0) (TD) (11.88–21.5 km/hr)	63.5 (47.4) (21.6 km · h−1 – 26.99 km/hr)	14.0 (± 3) (TD) (> 27 km/hr)	7.3 (0.8)
	
Clarke, 2017 [46]		33 (Senior = 22, Elite = 11)	Elite	Elite NA 1.69 ± 0.02 68.6 ± 4.4	12	1078 ± 197	85.8 ± 3.9	323 ± 87 (TD) (12.6 km/ hr – 17.99 km/hr)	120 ± 41 (TD) (> 18 km/hr)	148.6 ± 39.1 (TD)	8.05 ± 0.55
	
			Sub Elite	Sub Elite NA 1.70 ± 0.07 70.4 ± 9.3		1099 ± 228	98.2 ± 12.4	330 ± 97 (TD) (12.6 km/ hr – 17.99 km/hr)	102 ± 44 (TD) (> 18 km/hr)	126.9 ± 42.9 (TD)	7.40 ± 0.52
	
Couderc, 2023 [42]		18	Elite	24.2 ± 2.4 168 ± 4 67.6 ± 10.5	19		161 ± 19 (1 min peak)		66 ± 25 (TD) (> 16 km/hr)		8.0 ± 0.5
	
Goodale, 2017 [13]		20	Elite	24.0 ± 3.6 168.4 ± 6.0 69.0 ± 5.0	5	1352 ± 306	87 ± 11	59 ± 7 (0.7–12.59 km/hr)	16 ± 5 (12.6–17.99 km/hr)	7 ± 3 (18–23.4 km/hr)	6.9 ± 0.8
	
Hills, 2024 [99]		13	Elite	25 ± 5 175.0 ± 4 73.0 ± 4	6		135 ± 26		31.0 ± 12 (> 18 km/hr)		
	
Malone, 2018 [53]		27	Elite	24.4 ± 2.1 168.0 ± 7.1 67.9 ± 4.3	36	1652 ± 132	116.1 ± 9.4		14.2 ± 3.1 (15.84–19.79 km/ hr)	98–143 (TD) (> 19.8 km/hr)	7.69 (7.31–8.23)
	
Middeldine, 2018 [98]		12	Elite	25.6 ± 4.9 168.1 ± 5.0 65.5 ± 5.5	6	F = 1601 ± 192 B = 1527 ± 256	F = 97 ± 8 B = 98 ± 6		F = 277 ± 67 B = 234 ± 51 (14.4–20.99 km/hr) (TD)		7.19 ± 0.75
	
Muller, 2024a [48]		24	Sub Elite	16.88 ± 0.54 160.8 ± 6.66 62.69 ± 13.9	1	VSR = 1251.51 ± 285.77 BRA = 1356.49 ± 120.32	VSR = 99.80 ± 8.67 BRA = 106.41 ± 11.65	VSR = 28.48 ± 7.33 BRA = 29.73 ± 9.99 (8.1–14 km/h)	VSR = 6.02 ± 3.92 BRA = 8.61 ± 2.41 (14.1–18 km/hr)	VSR = 0.09 ± 0.16 BRA = 2.04 ± 2.81 (> 18 km/hr)	
	
Portillo, 2014 [56]		20 (Elite = 10, Sub-elite = 10)	Elite	Elite 26.27 ± 4.05 166.7 ± 6.7 65.39 ± 5.01	8	1642.2 ± 171.2		275.0 ± 87.5 (14.1–18 km/hr)	102.6 ± 48.2 (18.1–19.99 km/hr)	118.8 ± 61.4 (> 20 km/hr)	6.915 ± 2.5
	
			Sub Elite	Sub Elite 32.1 ± 6.4 167.4 ± 3.0 66.5 ± 5.4	1363.4 ± 221.8		199.4 ± 78.9 (14.1–18 km/hr)	46.2 ± 32.7 (18.1–19.99 km/hr)	47.0 ± 38.8 (> 20 km/hr)	6.19 ± 1.6
	
Sella, 2021 [101]		30	Sub Elite	22.0 ± 5.0 168.0 ± 0.05 69.0 ± 7.0	6	1123 ± 123.5		16.64 ± 23% (12.6–17.99 km/hr)	7.71 ± 20% (18–26.99 km/hr)	5.43 ± 1.55 (> 27 km/hr)	7.4 ± 6%
	
Suarez-Arrones, 2012 [55]		12	Elite	27.8 ± 4.0 165.5 ± 6.2 63.7 ± 4.8	5	1,556.2 ± 189.3		255.7 ± 88.3 (TD) (12.1–14.0 km/hr)	57.1 ± 40.8 (14.1–18.0 km/hr)	84.0 ± 64.8 (18.0–20.0 km/hr)	6.44 ± 0.61
	
Vescovi, 2015 [49]		Elite = 16, Sub-elite = 13	Elite	NA NA NA	10 (5, 5)	1468 ± 88 (1415–1521)	95 ± 5 (92–98)	36 ± 5 (33–39) (8.1–16.0 km/hr)	14 ± 3 (13–16) (16.1–20.0 km/hr)	8 ± 4 (6–11) (20.1–32.0 km/hr)	7.4 ± 0.53
	
			Sub Elite		1252 ± 135 (1170–1333)	91 ± 11 (84–97)	38 ± 12 (31–45) (8.1–16.0 km/hr)	10 ± 4 (7–12) (16.1–20.0 km/hr)	4 ± 3 (2–6) (20.1–32.0 km/hr)	6.83 ± 0.75

Cummins, 2021 [110]	Rugby League	80	Elite	NA NA NA			81–89.1 (2.6–4.3) (10–min epoch)				
	
Cummins, 2023 [66]		85	Elite	NA NA NA	6	F = 4087.4 ± 254.6 B = 5429.0 ± 135.3 A = 4961.6 ± 273.5	F = 76.2 ± 1.6 B = 76.6 ± 1.1 A = 79.0 ± 1.7	F = 12.1 ± 0.7 B = 12.0 ± 0.6 A = 13.5 ± 1.1 (11.5–17.49 km/hr)	F = 2.0 ± 0.2 B = 4.2 ± 0.3 A = 3.1 ± 0.4 (17.5–20.99 km/hr)	F = 0.4 ± 0.1 B = 1.6 ± 0.2 A = 0.9 ± 0.2 (> 21 km/hr)	
	
Cummins, 2024 [67]		172	Elite	NA NA NA		F = 4595.1–4908.7 B = 6021.1–6216.3 A = 5646.7–5884.5	F = 77.6–79.0 B = 76.2–77.8 A = 79.9–81.1		F = 2.2–2.4 B = 4.3–4.5 A = 2.4–2.6 (> 17.5 km/hr)	F = 0.4–0.6 B = 1.6–1.8 A = 0.6–0.8 (> 21 km/hr)	
	
Emmonds, 2020 [65]		58	Elite (International)	NA NA NA	9	F = 4680 ± 1618 B = 6016 ± 1263	F = 76.7 ± 6.1 B = 75.2 ± 8.8		F = 1.1 ± 0.9 B = 4.1 ± 4.8 (18–25.1 km/hr)	F = 0.0 ± 0.1 B = 0.2 ± 0.3 (> 25.2 km/hr)
	
			Elite (National)			F = 4737 ± 1596 B = 6099 ± 1883	F = 73.4 ± 10 B = 74.3 ± 9.1		F = 1.2 ± 1.8 B = 2.3 ± 1.7 (18–25.1 km/hr)	F = 0.0 ± 0.0 B = 0.1 ± 0.2 (> 25.2 km/hr)	
	
Newans, 2021 [63]		117	Elite	26.8 ± 5.4 1.68 ± 0.07 76.7 ± 11.9	4	2908–5504	75.7–82.7	7.6–7.7 (4.2–14.1) (12.01–15.00 km/hr)	2.9 (1.6–5.3) (15.01–17.99 km/ hr)	1.8–2.0 (1.0–3.7) (> 18 km/hr)	
	
Quinn, 2020 [64]		31	Elite	27 ± 5 NA NA	8	F = 6416 (5973–6859) B = 6710 (6281–7139) H = 7011 (6509–7512)		492–778 (12.01–15 km/hr) (TD)	265–722 (> 15 km/hr) (TD)	

* TD = Total Distance; B = Backs, F = Forwards, A = Adjustables; VSR = Vem Ser Rugby group; BRA = Brazilian youth rugby team;

The most commonly reported variables for match demands were absolute and relative distances covered, which were assessed using GPS devices (n = 7) (Table 5). Both elite and sub-elite athletes demonstrated a wide range of total distances covered during matches, with sub-elite athletes having greater variability in distance covered (Figure 3, Table 5). Relative distances also varied significantly between elite and sub-elite athletes. Elite athletes recorded higher average relative distances compared to sub-elite athletes (Figure 3).

Additionally, elite athletes covered greater distances at high and very-high speeds compared to sub-elite athletes (Table 5). For instance, elite athletes’ average distances at HSR ranged from 9.8 (± 3.6) to 28.0 (23.9 – 32.1) m/min [28], while sub-elite athletes ranged from 7.8 (± 4.5) [29] to 13.1 (± 3.6) m/min [30]. Similarly, at very-high speeds, elite athletes ranged from 2.61 (± 1.08) to 9.6 (7.3–11.9) m/min, whereas sub-elite athletes ranged from 2.74 (± 2.1) to 3.36 (± 1.4) m/min (Table 5). Finally, peak velocities for elite athletes ranged between 6.75 and 7.22 m/sec [31], while subelite athletes exhibited peak velocities between 6.67 and 6.94 m/sec [30] (Figure 3).

### Rugby Union

Nineteen rugby union studies were reviewed, with all of them reporting on the anthropometric traits of athletes (Table 3). Commonly reported variables included height, body mass, and body composition. Of these, five studies used dual-energy X-ray absorptiometry (DXA) to assess body composition [32–36], while three studies reported the sum of skinfolds as a measure of adiposity [34, 37, 38]. The most reported physical quality variables included 10-metre acceleration sprint times, 1RM front squat, and vertical jump tests (Table 4). Eleven studies described the physical qualities of rugby union athletes (Table 4). Elite athletes recorded higher average peak 10-metre sprint times, 40-metre sprint times, and countermovement jump heights compared to sub-elite athletes (Table 4).

Physical match demands were collected using GPS units (n = 7), as summarised in Table 5. Elite athletes covered average total distances between 3,240 m (± 287) [38] and 5,820 m (± 512) [39–41], with relative distances ranging from 62.1 (± 1.2) to 72.9 m/min [38] and peak relative distances of 155 (± 21) m/min over a 60-second period [42] (Figure 3). Sub-elite athletes covered average total distances between 4,982 m (± 917) [43] and 5,829 m (± 1,022) [12], with relative distances between 54.8 (± 9.1) [43] and 67.9 m/min (± 6.9) [39] (Figure 3).

HSR distances for elite athletes ranged from 16.8 to 46 m/min for speeds of 12.6–19.79 km/hr [38], with total distances from 105 m (± 74) [37] to 220 m (± 156) at speeds > 18.0 km/hr [39]. Average relative distances at very-high-speed running (VHSR) ranged from 0.3 to 3.8 m/min [38]. The peak velocity for elite athletes was recorded at 7.6 m/sec [42], while sub-elite athletes had peak velocities ranging from 5.83 to 6.5 m/sec [12, 39, 43, 44] (Figure 3).

### Rugby Sevens

Eighteen studies reported on female rugby sevens athletes, all provided data on anthropometric traits (Table 3), with several also reporting on physical qualities and match demands (Tables 4 and 5). Common anthropometric variables reported included height, body mass, and sum of skinfolds, with elite athletes generally presenting lower skinfold values than sub-elite athletes (86.8 mm ± 11.2 vs. 89 mm ± 20) [45–48]. Physical qualities such as 10-metre sprint times, Yo-Yo IRT1 scores, and 1RM strength measures were frequently assessed. Elite athletes typically demonstrated superior performance across these metrics, including faster sprint times (10 m: 1.76 s ± 0.05 vs. 1.82 s ± 0.06) and greater aerobic fitness (Yo-Yo IRT1: 1,702 m ± 252 vs. 1,058 m ± 249) [45, 46, 49, 50]. Strength measures were also greater elite athletes, with higher 1RM front squat values (84.5 kg ± 5.8 vs. 80.0 kg ± 17.0) [45, 47, 50–52] and relative squat scores (1.22 vs. 1.30) (Table 4).

Match demands were assessed using GPS and included total and relative distances, peak velocities, and high-speed running metrics. Elite athletes generally covered greater total distances (1,652 m ± 132 vs. 1,463 m ± 157) [46, 53, 54], higher relative distances (116.1 m/min ± 9.4 vs. 98.2 m/min ± 12.4) [46, 49, 53], and reached higher peak running velocities (8.05 m/s ± 0.55 vs. 7.4 m/s ± 0.52) [46, 55, 56] compared to sub-elite athletes (Figure 3, Table 5).

### Rugby League

Fourteen studies reported on female rugby league athletes, with nine providing data on anthropometric traits (Table 3). Commonly reported variables included height, body mass, and body composition, with three studies [57–59] using DXA and one reporting sum of skinfolds [60]. Physical qualities such as 10-metre and 40-metre sprint times, vertical jump height, and aerobic fitness were assessed across studies (Table 4). Elite athletes generally demonstrated superior performance, with faster sprint times (10 m: 1.87 s ± 0.09 vs. 2.04 s ± 0.1; 40 m: 6.13 s ± 0.25 vs. 6.59 s ± 0.41) [58, 60], higher vertical jump scores (35.7 cm vs. 27.9 cm) [57, 60–62], and greater aerobic capacity (Yo-Yo IRT1: 670 m ± 151.6) [60].${\dot{\text{V}}\text{O}}_{2\max}$ values were reported for elite athletes (40.0–47.2 mL/kg/min) [57, 59], with no data available for sub-elite groups.

Match demands were assessed using GPS technology and included total and relative distances, running velocities, and intensity bands (Table 5). Elite athletes covered greater total distances (7,011 m vs. 6,099 m) [63–65] and higher relative distances (81.1 m/min ± 1.7 vs. 74.3 m/min) [65–67], with peak 10-minute epochs reaching up to 89.1 m/min [66]. At higher running intensities, elite athletes recorded greater distances at high-speed and very-high-speed running compared to sub-elite athletes (HSR: 4.5 m/min ± 0.3 vs. 2.3 m/min; VHSR: 2.0 m/min vs. 0.1 m/min) [63, 66–68]. No studies reported peak velocity data for either group.

## DISCUSSION

This scoping review systematically examined the physical traits, and match demands of female athletes across four major collision sports: Australian football, rugby union, rugby sevens, and rugby league. Rugby union emerged as the most frequently studied sport, with Australian football being the least represented. Across studies, height and body mass were the most commonly reported anthropometric metrics, with eight studies using skinfold measurements and another eight employing DXA technology for more detailed body composition analysis. The most frequently assessed physical qualities included sprint speed (10 m and 40 m sprints), vertical jump height, and maximal strength (1RM bench press and squat variations). Aerobic fitness and repeated high-intensity efforts were commonly evaluated using the Yo-Yo IRT1 and 1,600 m shuttle tests. Match demands were primarily captured using GPS technology, with total distance, relative distance, HSR, VSHR, and peak velocity being the most reported metrics. Notably, elite athletes consistently demonstrated superior physical and performance characteristics compared to their sub-elite counterparts, particularly in speed, strength, and aerobic capacity. These findings provide a foundation to support evidence-based training strategies and talent identification practices tailored to the specific demands of each sport and playing level.

**FIG. 2:**
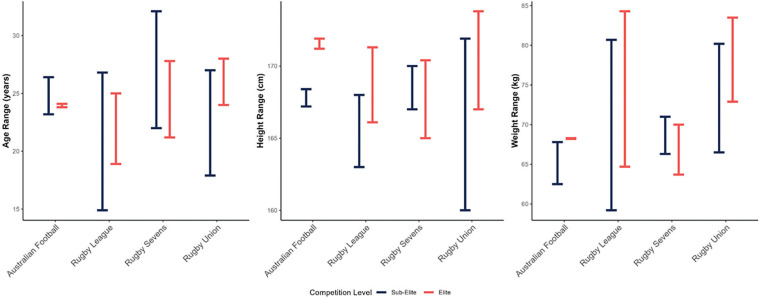
Anthropometric traits of female collision sport athletes

Across all four collision sports, sub-elite athletes had a greater age variability compared to elite athletes (Figure 2), likely reflecting broader age classifications or extended developmental windows. Elite athletes were generally taller and heavier than sub-elite athletes, suggesting these anthropometric traits are key in playing level attainment. This aligns with previous research in male Australian football athletes [69–71].

**FIG. 3:**
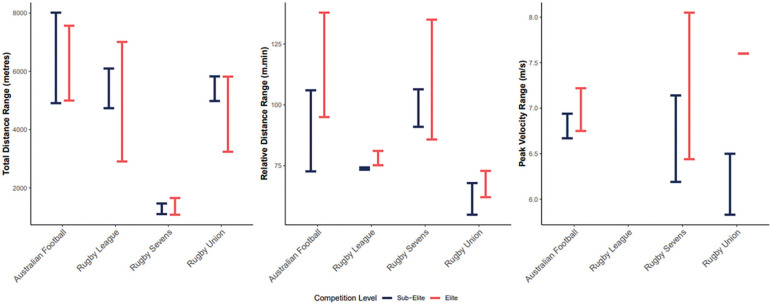
Running demands of female collision sport athletes

In one study examining sub-elite Australian football athletes, those selected for competition demonstrated superior aerobic capacity, as reflected by greater distances achieved in the Yo-Yo IRT1 test [11]. Athletes with higher Yo-Yo IRT1 scores have also been shown to exhibit greater high-speed running ability and a more demanding activity profile compared to those with lower high-speed running capacity [72]. The Yo-Yo IRT1 test focuses on the capacity to perform intermittent exercise leading to maximal activation of the aerobic system [73]. Aerobic capacity is known to be associated with match influence, quantified by the number of ball possessions (e.g., kicks, handballs) and participation in contested and uncontested plays per minute, making it an important attribute for athlete selection and performance [74]. Wijekulasuriya et al (2024), investigating sub-elite athletes, reported an average maximal velocity of 7.22 m/s [75], further highlighting the importance of HSR capabilities in this cohort.

While total distances covered were similar between elite and subelite Australian football athletes (Table 5), elite players achieved higher relative distances (m/min) and greater HSR, VHSR, and peak velocities within a game (Table 5) [11, 29–31, 76–78]. These findings highlight the greater intensity of elite-level match play and are consistent with male data, where higher running speeds and repeat sprint ability differentiate playing levels [74]. Understanding the relative distances covered and running demands can help inform training strategies and optimise physical preparation by tailoring conditioning programs to reflect the intensity and duration of match play. This allows practitioners to progressively expose athletes to gamelike workloads, ensuring they are adequately prepared for competition demands while also managing fatigue and reducing injury risk through appropriate load monitoring and recovery planning.

In rugby union, elite female athletes were consistently taller and heavier than their sub-elite counterparts (Table 3), underscoring the importance of body mass in meeting the physical demands of the sport. Specifically, adequate body mass in the form of lean mass, fat mass, and bone mass appears to be crucial for withstanding the frequency and intensity of collisions during offensive and defensive match-play [79, 80]. While fat mass may contribute to cushioning during contact, lean mass supports the development of power, speed, and aerobic fitness, all of which are essential for high-level performance [81–83]. These characteristics are particularly important for forwards, who experience higher collision loads and require greater mass and strength to dominate physical contests [84]. Interestingly, no significant differences were found in 10 m and 40 m sprint performance between playing levels [38, 85]. This may reflect the limited number of studies (n = 5) reporting these outcomes or variability in testing protocols. Alternatively, it may suggest that sprint speed alone is not a strong differentiator of playing level in rugby union, particularly when compared to other physical qualities such as strength and power.

Rugby union athletes must possess considerable strength and power to manage frequent mauls and rucks, despite position-specific variations in force and power characteristics [86–91]. Elite athletes displayed higher 1RM values in bench press and front squat tests than sub-elite players (Table 4), supporting the idea that strength may be a more distinguishing factor than sprint speed, especially for forwards. Position-specific qualities such as acceleration and mass also influence sprint momentum, calculated as the product of an athlete’s mass and sprint velocity, which is a key factor in tackle success and collision dominance. However, although greater momentum theoretically offers an advantage, there is limited empirical evidence that clearly separates its effects from those of technical or tactical skills [34, 85, 92].

Interestingly, elite rugby union athletes covered less absolute distance than sub-elites, possibly due to differences in match duration or substitution strategies. However, elite athletes recorded greater relative distances and higher peak velocities (Table 5; Figure 3). These findings are consistent with a recent scoping review [6], which noted increased match demands at elite levels in both rugby sevens and rugby union. In contrast to other sports, elite rugby sevens athletes had lower body mass than sub-elites, with similar heights across levels (Table 3, Figure 2). This contrasts with previous findings in male rugby sevens [93], male and female rugby union [94, 95] and male and female rugby league [62, 96], where clear anthropometric differences are more pronounced between positional groups across playing levels.

Rugby sevens, characterised by its shorter match duration and reduced number of players, places greater demands on intermittent high intensity running, requiring superior aerobic capacity and repeated sprint ability [97, 98]. Consequently, sevens athletes often exhibit higher relative lean mass and superior performance in the Yo-Yo IRT1 test [45, 47, 49]. Elite rugby sevens players also recorded significantly higher Yo-Yo IRT1 distances than sub-elite players, reflecting their greater aerobic fitness. Notably, rugby sevens athletes also covered greater relative distances (measured in metres per minute) compared to athletes in other collision sports, further underscoring the intense physical demands of the format [99, 100]. These findings suggest that enhanced aerobic capacity and the ability to sustain high-intensity efforts are key physiological characteristics that distinguish elite from sub-elite athletes in rugby sevens [50].

Elite athletes exhibited faster moderate-, high-, and very-high running speeds, as well as greater maximum velocity and maximum speed momentum than sub-elite athletes (Figure 3, Table 5), aligning with findings from a recent systematic review [101]. Caution is warranted when interpreting VHSR running efforts, as speed band thresholds varied considerably across studies (18 – 27 km/h). These inconsistences limit direct comparisons not only between playing levels within a sport, but also across different collision sports included in this review. Nonetheless, elite athletes consistently reached higher peak velocities than their sub-elite counterparts, reflecting a trend observed across all collision sports (Table 5). Speed is a critical physical characteristic for rugby sevens athletes, as it correlates with the number of line breaks and defenders beaten per match [93].

In rugby league, elite female athletes were also heavier than subelites, a trend mirrored in both rugby union and Australian football and supported in the male athlete literature [96, 102, 103]. Additionally, elite female rugby league athletes exhibited faster sprint times over 10 m and 40 m distances (Table 4), reinforcing speed as a distinguishing characteristic, particularly among backs who engage in more HSR and VHSR [103, 104]. Speed as a key distinguishing physical factor has also been found in research examining profiles of male rugby league athletes [10].

While previous research in male rugby league athletes has shown inconsistent relationships between vertical jump performance and playing standard, likely due to methodological and sampling variability [103, 105–109], the current review identified clearer trends in female cohorts. Elite athletes in rugby league and rugby sevens consistently demonstrated greater jump heights than sub-elite players (rugby league; 35.7 cm vs. 27.9 cm) [57, 60–62] (Table 4), suggesting that vertical jump may be a useful indicator of lower-body power in these sports. However, given the variability in testing protocols and sample characteristics across studies, jump height should be interpreted with caution and used alongside other performance metrics. For practitioners, this highlights the importance of incorporating comprehensive assessments, such as force-time profiling, to better capture the neuromuscular qualities that underpin elite performance in collision sports.

Both elite and sub-elite rugby league athletes recorded similar absolute distances (Table 5; Figure 3). However, relative distance was a more sensitive metric in this context. Only one study on female rugby league players [110] analysed peak locomotor demands using fixed and rolling time windows. Future research should incorporate ball-in-play analysis to better capture the specific running loads encountered in match play across female collision sports.

To date, no studies have reported on match demands of sub-elite female rugby league athletes. Only one study [65] comparing national and international players indicated that elite athletes covered greater distances at higher intensities (Table 5, Figure 3). However, variation in speed band thresholds across studies continues to hinder the ability to compare data meaningfully. Notably, no studies reported peak velocity for either elite or sub-elite female rugby league players (Figure 3). Given the importance of running speed for backs in rugby league [103, 104], future studies should prioritise the inclusion of peak velocity and determine match demands across playing levels and positions.

This scoping review highlights notable differences in physical and performance metrics, as well as match demands, between elite and sub-elite athletes in female collision sports such as Australian football, rugby union, rugby sevens, and rugby league. Elite athletes consistently demonstrate superior speed, strength, and aerobic capacity, key determinants of performance and selection. These findings offer practical guidance for coaches and performance staff to support the design of targeted training programs that address the specific developmental needs of sub-elite athletes. For example, the enhanced body composition, speed, and strength observed in elite rugby union players underscore the importance of progressive resistance training and speed development protocols. In rugby sevens, the emphasis on aerobic capacity and repeated sprint ability supports the use of high-intensity interval training to improve these qualities.

Understanding physical match demands, such as the greater distances covered at higher velocities by elite athletes, can inform the design of sport-specific conditioning drills that replicate game intensity and movement patterns. Importantly, emerging evidence also suggests a link between physical qualities and defensive effectiveness, such as tackle success and repeat-effort capacity, further reinforcing the value of physical development in improving both offensive and defensive outcomes [111]. Integrating these insights into training not only enhances performance but also may reduce injury risk by ensuring athletes are more optimally physically prepared for the demands of elite competition. Therefore, a comprehensive understanding of the physical and performance demands in female collision sports is essential for preparing athletes to compete at the highest level.

The findings of this scoping review are subject to several limitations. Firstly, there was considerable heterogeneity in how match demands and physical characteristics were defined and measured. This was particularly evident in the use of GPS technology, where differences in device brands introduced variability in reported metrics such as HSR and sprint distances. These discrepancies stem from differences in sampling rates, proprietary algorithms, and data processing methods. Additionally, inconsistencies in the use of absolute versus individualised thresholds for metrics like HSR and sprinting further complicate comparisons. These methodological issues not only limit comparability between playing levels within a sport but also across different collision sports included in this review. Greater transparency and standardisation in GPS-based data collection and reporting are essential to improve the reliability of future research.

Moreover, many studies did not control for confounding variables such as age, injury history, and playing position, which may have influenced the observed outcomes. The lack of standardised reporting on these variables further complicates interpretation. Most studies were cross-sectional, limiting insights into the longitudinal development of physical characteristics and match demands. Longitudinal research would be valuable in understanding how these attributes evolve over time, particularly in response to training interventions. Furthermore, several studies had small, homogeneous sample sizes, often restricted to specific sports or competition levels, which limits generalisability. The potential for publication bias, where studies with positive findings are more likely to be published, may also skew the overall understanding of physical demands in female collision sports. Future research should address these gaps by incorporating larger, more diverse samples, controlling for contextual variables, and employing robust, longitudinal designs. Standardising measurement techniques and improving methodological rigor will enhance comparability and lead to more meaningful conclusions.

## CONCLUSIONS

This scoping review identified 59 studies examining the physical qualities and match demands of athletes in four major team-based collision sports commonly played in Australia. Key physical attributes, such as faster sprint times, greater maximal strength (e.g., 1RM bench press and squat), and enhanced aerobic capacity (e.g., Yo-Yo IRT1 performance), consistently distinguished elite athletes from their sub-elite counterparts. However, the risk of bias assessment revealed that confounding variables such as sleep, nutrition, and training load were not consistently controlled or reported. Additionally, the use of different GPS technologies and inconsistent threshold definitions for performance metrics introduced significant variability, limiting comparability both within and across sports. These methodological limitations may influence the interpretation of how physical qualities impact match performance. Future research should prioritise methodological consistency by standardising GPS metrics, clearly defining performance thresholds, and controlling for contextual variables such as playing position and match phase. Longitudinal studies using validated and reliable tools are also needed to understand how physical qualities develop over time and influence performance outcomes.

Despite these limitations, the findings offer valuable insights for practitioners. Specifically, greater lower-body strength, aerobic capacity, and sprint performance appear to be key differentiators of elite-level performance in female collision sports. These attributes can inform talent identification processes, guide the design of position-specific conditioning programs, and support the development of evidence-based training interventions tailored to the demands of each sport and playing level.
